# Increased Diabetes Complications in a Mouse Model of Oxidative Stress Due to ‘Mismatched’ Mitochondrial DNA

**DOI:** 10.3390/antiox13020187

**Published:** 2024-02-01

**Authors:** Andrzej S. Januszewski, Rachel Blake, Michael Zhang, Ben Ma, Sushma Anand, Carl A. Pinkert, Darren J. Kelly, Alicia J. Jenkins, Ian A. Trounce

**Affiliations:** 1 Department of Medicine, St. Vincent’s Hospital, University of Melbourne, Fitzroy, VIC 3065, Australia; andrzej.januszewski@sydney.edu.au (A.S.J.); michael.zhang@pharmalegacy.com (M.Z.); dentbenma@gmail.com (B.M.); darrenjk@unimelb.edu.au (D.J.K.); alicia.jenkins@baker.edu.au (A.J.J.); 2 NHMRC Clinical Trials Centre, The University of Sydney, Sydney, NSW 2006, Australia; 3 Sydney Pharmacy School, The University of Sydney, Sydney, NSW 2006, Australia; 4 Centre for Eye Research Australia, Royal Victorian Eye and Ear Hospital, Melbourne, VIC 3002, Australia; rachel.phillips@acu.edu.au (R.B.); sushma.anand@unimelb.edu.au (S.A.); 5 Ophthalmology, Department of Surgery, University of Melbourne, Melbourne, VIC 3000, Australia; 6 Department of Pathobiology, College of Veterinary Medicine, Auburn University, Auburn, AL 36849, USA; cap@auburn.edu; 7 Baker Heart and Diabetes Institute, 75 Commercial Road, Melbourne, VIC 3004, Australia

**Keywords:** diabetes, diabetes complications, heart, kidney, mouse strain, mtDNA, mitochondria, oxidative stress, xenomitochondrial mouse

## Abstract

Associations between chronic diabetes complications and mitochondrial dysfunction represent a subject of major importance, given the diabetes pandemic and high personal and socioeconomic costs of diabetes and its complications. Modelling diabetes complications in inbred laboratory animals is challenging due to incomplete recapitulation of human features, but offer mechanistic insights and preclinical testing. As mitochondrial-based oxidative stress is implicated in human diabetic complications, herein we evaluate diabetes in a unique mouse model that harbors a mitochondrial DNA from a divergent mouse species (the ‘xenomitochondrial mouse’), which has mild mitochondrial dysfunction and increased oxidative stress. We use the streptozotocin-induced diabetes model with insulin supplementation, with 20-weeks diabetes. We compare C57BL/6 mice and the ‘xenomitochondrial’ mouse, with measures of heart and kidney function, histology, and skin oxidative stress markers. Compared to C57BL/6 mice, the xenomitochondrial mouse has increased diabetic heart and kidney damage, with cardiac dysfunction, and increased cardiac and renal fibrosis. Our results show that mitochondrial oxidative stress consequent to divergent mtDNA can worsen diabetes complications. This has implications for novel therapeutics to counter diabetes complications, and for genetic studies of risk, as mtDNA genotypes may contribute to clinical outcomes.

## 1. Introduction

The prevalence of Type 1 and Type 2 diabetes is increasing globally, with ≈80% of people with diabetes living in disadvantaged regions [1]. The personal and socioeconomic costs are substantial, particularly when chronic disease complications develop [1]. Despite an array of modern glucose, lipid and blood pressure drugs and technologies such as insulin pumps and continuous glucose monitors, many people with diabetes develop chronic complications, such as diabetic retinopathy, nephropathy and cardiovascular disease, including cardiomyopathy [1]. Globally, diabetes is a common cause of vision loss, kidney failure and heart failure. Additional therapies targeting key pathways in the pathogenesis of diabetes-related tissue damage and protection may be of clinical value. Decades ago, Brownlee et al. [2] proposed a unifying hypothesis implicating increased mitochondrial oxidative stress as a common downstream mediator of well-accepted hyperglycemia-mediated promoters of chronic complications, activated protein kinase C, advanced glycation end-product (AGE) formation, and the polyol and hexosamine pathways [3,4].

Basic science studies demonstrated that therapeutics that reduced mitochondrial oxidative stress ameliorate cellular and tissue damage [2,3]. Almost two decades later, ongoing basic and clinical research confirms and extends the importance of mitochondrial dysfunction in the pathogenesis of many human diseases, including diabetic cardiomyopathy [5,6,7,8] and diabetic nephropathy, including glomerular, tubular and interstitial damage [9,10,11,12]. Of relevance and of major importance, there have also been advances in mitochondria-targeted therapies, be they pleiotropic effects of repurposed drugs, such as metformin, which promotes mitochondrial biogenesis, and was renoprotective in high cardiovascular disease risk adults with Type 1 diabetes [13], and high dose Co-Enzyme Q, which may improve outcomes in patients with heart failure [14]. A growing list of drugs show promise in different mitochondrial diseases, some of which may be of therapeutic value in metabolic diseases, including diabetes [15].

Mitochondria are key to addressing oxidative stress. In keeping with a key role of mitochondria number and/or function in diabetes and its outcomes, we recently reported lower mitochondrial DNA copy number (mtDNA-CN) in blood of adults with Type 1 diabetes relative to their non-diabetic peers, and in diabetic subjects with vs. without kidney damage [16], as noted in people with Type 2 diabetes [17]. Other studies demonstrated lower mtDNA-CN with increasing age and associations with health status [18].

Studies in well-characterized animal models are a useful precursor to human clinical trials. Trounce and Pinkert [19,20] developed a genetically modified mouse model, the ‘xenomitochondrial (XM) mouse’ that has an introduced defect in the mitochondrial respiratory chain consequent to ‘mismatched’ nuclear and mtDNA [21]. This model has not previously been assessed in the setting of diabetes, and we do so herein. To determine whether the XM mouse is a suitable model for the study of diabetes and some of its chronic complications, we characterize cardiac and renal structure and function in diabetic and non-diabetic XM and wild type (WT) mice. We hypothesize that relative to non-diabetic WT mice oxidative stress and AGEs will be increased in XM mice and even more so in diabetic XM mice, illustrating characteristic renal and cardiac damage.

## 2. Research Design and Methods

The project was approved by the St Vincent’s Hospital Melbourne Animal Research Ethics Committee (Approval 009/08 ‘Mitochondrial oxidative stress and diabetic complications’).

### 2.1. Xenomouse

The production of the xenomitochondrial mouse (XM) has been detailed previously [19,20]. The mouse harbors the mitochondrial genome of the Indian native field mouse *Mus terricolor*, while the nuclear genome is that of the C57BL/6J mouse due to backcrossing XM females with C57BL/6J males for over 25 generations. Thus, the nuclear genes of the WT and XM are essentially identical, while the mtDNAs differ considerably. Since mtDNA encodes 13 protein subunits of the oxidative phosphorylation complexes (OXPHOS), the rationale for producing the mouse was to investigate how ‘mismatched’ mtDNA and nuclear genes may impact disease phenotypes via subtle impacts on OXPHOS, therefore modelling the diversity of mtDNA genotypes in the human population. At young ages, the XMs demonstrate no overtly different phenotypes.

### 2.2. Induction of Diabetes and Hyperglycaemia

Mice were housed according to standard animal care protocols that complied with animal care procedures from the Australian code for the care and use of animals for scientific purposes [22] and were approved by the animal ethics committee of St. Vincent’s Hospital Melbourne (Australia). Wild type (WT) mice (C57BL/6J from SVHM BioResources Centre stock) and the XM mice were maintained in a 22 ± 1 °C, 12 h light (40 lux, on at 8 am)/12 h dark environment and were provided with murine chow and water ad libitum.

To mimic diabetes patient treatment regimens, particularly for Type 1 diabetes where exogenous insulin delivery is essential for life, we treated diabetic mice with low doses of insulin over the period of hyperglycemia. Following fasting for four hours, 8-week-old mice were injected intraperitoneally with streptozotocin prepared to a concentration of 5.5 mg/mL in sterile PBS. The solution was prepared immediately prior to injection to avoid degradation of streptozotocin. To ensure extensive pancreatic beta cell destruction, a single large dose of streptozotocin was administered intraperitoneally to a final concentration of 200 mg/kg body weight. When determined to be hyperglycemic (after seven days), mice were administered 10 U of insulin (Actrapid, Novo Nordisk, Copenhagen, Denmark) daily via intraperitoneal injection. This was maintained for 20 weeks, during which time the weight of all animals and blood glucose levels (BGL) of all diabetic animals were tested and recorded weekly.

Some controversy exists around the single high dose streptozotocin model used in the present study, compared with repeated low-dose streptozotocin models, with non-specific toxicity being a potential confounding factor [23]. When compared, the lower streptozotocin model can also produce undesirable effects such as higher inflammatory markers in muscles [24]. We chose the high streptozotocin model, as it ablates all pancreatic beta cells, resulting in insulin dependent diabetes, to mimic human insulin dependent diabetes. Additionally, slower progression to diabetes can introduce variability among subjects in terms of the onset and severity of diabetes. This approach may also require more prolonged observation and care of the animals. Multiple small doses, though potentially less immediately harmful, involve prolonged periods of stress and handling for the animals.

### 2.3. Blood and Tissue Samples Collection and Analysis

Post echocardiography and cardiac catheterization, 500 μL of blood was collected in heparinized syringes from the inferior vena cava. An aliquot of the whole blood was preserved for future analyses (such as glycated hemoglobin), while the rest was centrifuged, and the plasma fraction was stored at −80 °C for later plasma biochemistry examination. Animals were euthanized by CO_2_ inhalation followed by cervical dislocation. Kidneys were excised, decapsulated and sliced transversely. Half of the kidney was snap frozen with liquid nitrogen and the other half fixed with 10% neutral buffered formalin (NBF; 30 mM NaH_2_PO_4_·H_2_O; 46 mM NaH_2_PO_4_, 40% formaldehyde) and paraffin-embedded for subsequent histological analyses. The heart was excised and sliced transversely into three sections, of which the middle section was preserved with 10% NBF and paraffin-embedded for subsequent histological analyses. Skin collected from the dorsal part of the animal was collected and snap frozen with liquid nitrogen for future analysis.

Blood glucose levels were quantified using a portable glucose meter. HbA1c levels and quantified as glycated hemoglobin by HPLC using boronate affinity column [25], and results converted to HbA1c using linear regression formula HbA1c (%) = 0.587 × (GHb (%)) + 1.720. All runs included diabetic and non-diabetic mouse samples.

### 2.4. Histology

Five-micron sections were stained with haematoxylin and eosin (H&E), picrosirius red, or anti-nitrotyrosine antibodies, as described below.

#### 2.4.1. H&E

In heart and kidney, general morphological changes were assessed by staining with H&E. After dewaxing (with histolene) and dehydration (100% to 90% ethanol), sections were incubated in hematoxylin (stains cell nuclei). Ammonia water was used to blue the haematoxylin stain. Sections were then incubated with eosin, which stains cytoplasmic contents. Following further dehydration with ethanol and clearing with histolene, coverslips were applied using distyrene plasticizer xylene (DPX).

#### 2.4.2. Picrosirius Red

Renal fibrosis was analyzed by staining for collagen using picrosirius red after dewaxing (with histolene) and dehydration (serial dilutions of ethanol), sections were incubated in picrosirius red for 1 h at room temperature. Then, 1% acetic acid was used to wash the slides. Following further dehydration with ethanol and clearing with histolene, coverslips were applied using DPX. To determine the extent of extracellular matrix deposition in the mid left ventricle, images were captured of the picrosirius red stained sections. An area of red sub-endocardium was selected for its color range and the proportion of matrix deposition was calculated using image analysis with ImageJ (version 2.0) [26] and described below.

#### 2.4.3. Nitrotyrosine Immunohistochemistry

Oxidative damage in heart and kidney tissue was determined by immunohistochemical staining with an anti-nitrotyrosine antibody. Nitrotyrosine is a marker of NO-dependent, reactive nitrogen species-induced nitrative stress. After dewaxing (with histolene) and dehydration (in serial dilutions of ethanol), sections were washed with tap water. Sections were incubated with 3% hydrogen peroxide for 10 min at room temperature to block exogenous peroxidase activity. The sections were then washed twice in 1× PBS. In order to block non-specific protein binding, the sections were incubated for 20 min with 1:10 dilution of normal goat serum (NGS) in 1× PBS and then incubated with rabbit anti-nitrotyrosine antibodies (Millipore, Bedford, MA, USA) (1:400 dilution) overnight (18 h) at 4 °C. Sections were then washed in 1× PBS (3 × 5 min washes) and incubated with goat anti-rabbit horseradish peroxidase (Millipore) for 45 min at room temperature, then rinsed with 1× PBS (2 × 5 min). Antibody binding was then visualized with 3,3′-diaminobenzidine tetrahydrochloride (DAB) as a chromogen for 1–3 min (under control with light microscope), rinsed in tap water for 5 min and counterstained in Mayer’s hematoxylin. Following differentiation in Scott’s tap water, further dehydration with ethanol and clearing with histolene, coverslips were applied using DPX. Sections incubated with 1:10 NGS (instead of primary antiserum) served as negative controls. Positive controls were generated by incubating sections for 20 min in 1 mM sodium nitrite and 1 mM H_2_O_2_ in 100 mM sodium acetate after being de-paraffinized.

To determine the accumulation of nitrotyrosine in the cardiac and renal tissue, images were taken of the immunostained sections and analyzed using Image J, as described below.

### 2.5. Analysis of Histology and Immunostaining

#### 2.5.1. Picrosirius Red Stain Analysis Using ImageJ

Image J software [27] was used to quantify the red-stained collagen in an image of kidney or heart tissue section stained with picrosirius red as described by [26]. The scale of the image was first converted to micrometers rather than pixels by calibrating to the scale bar and setting the scale to the appropriate length. The image was then split into three color channels (RGB stack), showing the best separation of color in the green channel. The green channel was selected, and the upper limit of the color threshold was adjusted so that the red-stained collagen was highlighted. Through optimization, this threshold was established as 75% of the automatically determined maximum. The area of picrosirius red stained was expressed as a proportion of the total area of the image. An automatic analysis of these images using the method described above was adapted from [26].

#### 2.5.2. Nitrotyrosine Immunostaining Analysis Using ImageJ

To quantify the nitrotyrosine immunostaining in the heart and kidney, the proportion of brown staining was calculated using the image analysis software ImageJ. As described in the previous section, the image was split into three color channels (RGB) and the image showing the best color separation was chosen. However, in the grayscale spectrum, the brown stain of the nitrotyrosine immunostaining is close to the haematoxylin staining of the nuclei, and therefore must be eliminated from the analysis. As the dark nitrotyrosine staining is unable to be separated from the nuclei staining, two measures must be made. Firstly, to measure nuclei staining plus nitrotyrosine staining, the blue channel was chosen. The upper limit of the threshold was established to be 90% of the default threshold. Next, the red channel was used to detect the nuclei specific staining and an upper threshold limit of 80% of the automatic threshold was used. The area of nitrotyrosine-specific staining was calculated by subtracting the red channel nuclei-specific staining (total area) from the blue channel staining (total area) due to nuclei and nitrotyrosine staining.

### 2.6. Skin Advanced Glycation End-Products

Mouse skins were firstly de-furred and dried. About 50 mg of skin sample was then transferred into chromerge-treated 13 × 100 mm screw cap tubes and reduced with 0.1 M NaBH_4_ freshly reconstituted with 0.1 M sodium borate buffer (pH 9.1) for 4 h at room temperature or 16 h at 4 °C. Subsequently, samples were washed twice with D.I. water. After the addition of 40 μL internal standard mixture consisted of 264 nmol of d_8_-Lys (CDN Isotopes, Pointe-Claire, QC, Canada), 3.18 nmol of d_2_-CML (PolyPeptide Laboratories, Strasbourg, France) and 2.66 nmol of d_8_-CEL (generous donation from J.W. Baynes from University of South Carolina, Columbia, SC, USA), samples were hydrolyzed with 4 mL 6 M HCL at 110 °C for 24 h. Hydrolysates were then dried in vacuo and reconstituted with 2 mL of D.I. water. An aliquot of 1 mL was preserved for pentosidine analysis with HPLC, while TFA was added to the rest to obtain a concentration of 1%.

For carboxymethyllysine (CML) and carboxyethyllysine (CEL) quantification amino acids were converted to their trifluoroacetyl methyl ester derivatives and analyzed by GC/MS as described [28,29] with modifications. Briefly, 4 mg of protein was reduced with 100 mM NaBH_4_ (freshly made) in 0.1 M sodium borate buffer (pH 9.1) for 4 h at room temperature or 16 h at 4 °C. Protein was precipitated with 10% (*w*/*v*) TCA followed with incubation for 5 min on ice. After centrifugation at 2000× *g* for 5 min at 4 °C, supernatant was discarded and the pellet was re-suspended in 500 µL of 0.1 N NaOH with brief ultrasound sonication. Internal standard mixture which consisted of 132 nmol of d_8_-Lys, 1.59 nmol of d_4_-CML and 1.33 nmol of d_8_-CEL was then added before samples were hydrolyzed with 6 N HCl for 24 h at 110 °C. Hydrolysates were dried in vacuo and re-suspended in 1 mL of 1% TFA before undergoing column extraction. Sep-Pak columns were activated with 4 mL methanol and washed with 4 mL 1% TFA. Samples were then transferred to prepared columns and eluate was collected in borosilicate 13 × 100 mm screw cap tubes. The column was subsequently washed with additional 3 mL of 1% TFA and eluate collected. Samples were dried in vacuo and methyl esters were prepared by addition of 1 mL of 1 M methanolic HCl (28 mL of anhydrous methanol + 2 mL acetyl chloride) followed by heating at 65 °C for 45 min. The methyl esters were dried again and converted to trifluoroacetyl derivatives by addition of 1 mL TFAA and incubating at room temperature for 1 hr. The derivatized samples were dried and reconstituted in 125 µL methylene chloride. Following centrifugation at 10,000× *g* for 5 min, supernatant was transferred to GC autosampler vials. Then, 2 µL was injected for GC/MS analysis.

GC/MS analyses were performed on a Shimadzu (Kyoto, Japan) model QP2010C gas chromatograph/mass selective detector equipped with a Shimadzu AOC-20i autosampler. A 30 m Zebron ZB-5 (crossband 5% diphenyl—95% dimethyl polysiloxane) capillary column (Phenomenex, Torrance, CA, USA) was used for GC separation. For GC/MS analysis, the injection port was maintained at 275 °C, MS interface at 230 °C and ion source temperature was 200 °C. The temperature program was: initial temperature 130 °C for 2 min, 4 °C/min ramp to 210 °C, 20 °C/min to 290 °C, then hold at 290 °C for 3 min.

The 392, 396, 379, 387, 180 and 187 ions were used for detection and quantification of CML, d_4_-CML, CEL, d_8_-CEL, Lys and d_8_-Lys, respectively, by selected ion monitoring (SIM) GC/MS. The levels of CML, CEL and Lys were determined, from the ratio between the areas under the representative peaks of the natural compound and its heavy labeled derivative, by external standardization utilizing standard curves generated from solutions with various concentrations of the six analytes. The amounts of CML and CEL were than normalized to the Lys content of the sample.

The reconstituted hydrolysates preserved during the sample preparation for GC/MS analysis of skin CML and CEL were filtered with sterile 0.45 μm filter (Millipore, Bedford, MA, USA) before heptafluorobutyric acid (HFBA; Sigma-Aldrich, St. Louis, MO, USA) was added to obtain a final HFBA concentration of 1%. For HPLC analysis of pentosidine, 50 μL of sample was injected in the HPLC. Reversed phase high performance liquid chromatography (RP-HPLC) was used for the measurement of pentosidine content of the sample. Chromatography was performed with Waters 474 scanning fluorescence detector in conjunction with Waters 600 controller and 717-plus auto-sampler (Waters, MA, USA) using Waters Atlantis dC_18_ 4.6 × 250 mm column with 5 µm particle diameter; flow rate was 1.2 mL/min, respectively. Three solvents were utilized for the gradient system: solvent A—0.1% HFBA in D.I. water, solvent B—50% acetonitrile (Merck, Darmstadt, Germany): 50% 0.1% HFBA, solvent C—100% acetonitrile. The solvent gradients were: 0 min = 90% A and 10% B, 10 min = 60% A and 40% B, 26 min = 50% A and 50% B, 31 min = 25% A and 75% B, 33 min = 100% C, 50 min = 90% A and 10% B, and held for at least 10 min. Subsequently, an injection of 55 μL pentosidine-spiked sample solution, 50 μL of the same sample spiked with 5 μL of 1% HFBA containing 0.5 pmol pentosidine (Case Western University, Cleveland, OH, USA), was analyzed with the same program in order to identify the peak representing pentosidine.

### 2.7. Renal Assessment

On the day before sacrifice, mice were placed into metabolic cages (Techniplast, Buguggiate, VA, Italy) for 24 h. Mice had access to a known amount of water and food. At the end of the caging period, water and food consumption was recorded, as well as urine output. Urine was collected and stored at −80 °C until required for analyses. Renal function was assessed by urinary albumin and creatinine levels determined by immunoturbidimetric assay (urinary albumin) and kinetic colorimetric assay based on Jaffè method (urinary creatinine) on Cobas Integra 400+ clinical chemistry analyzer (Roche, Basel, Switzerland). Plasma creatine was measured using a similar kinetic colorimetric assay based on the Jaffè method on the same analyzer.

### 2.8. Cardiac Assessment

#### Cardiac Function

Echocardiography, including Doppler examination, was performed using a Vivid 7 Dimension (GE Vingmed, Horten, Norway) echocardiograph with a 10 MHz phased array probe. Electrocardiographic data were acquired simultaneously. End-diastole was defined as the peak of the R wave, and end-systole was defined as the end of the T wave.

Animals were anaesthetized with pentobarbitone sodium 60 mg/kg intra peritoneal injection. Animals underwent echocardiographic interrogation in the left recumbent position. M-mode echocardiography was performed using a parasternal short axis view at the level of the papillary muscles. Left ventricular posterior (LVPWd) and anterior wall thickness (LVAWd) were obtained during diastole (d) and systole (s), as were the left ventricular internal diameter at end-diastole (LVIDd) and end-systole (LVIDs). Fractional shortening (FS) was then calculated according to the formula:

FS% = [(LVIDd − LVIDs)/LVIDd]) × 100

Left ventricular end-diastolic (EDV) and end-systolic volumes (ESV), obtained from the para-sternal long axis view were calculated according to a single plane area-length method [30].

All Doppler spectra were recorded for 10 cardiac cycles at a sweep speed of 200 mm/s. All parameters were assessed using an average of three beats, and calculations were made in accordance with the American Society of Echocardiography guidelines [30]. Data were acquired and analyzed by a single masked observer using EchoPAC (GE Vingmed) offline processing.

Post echocardiography, animals were placed on a warming pad (37 °C), intubated using a 14 gauge catheter, and ventilated using positive pressure with a tidal volume of 10% body weight at 70 breaths per minute using room air post echocardiography. Animals were secured in a recumbent position and the right jugular vein was cannulated with 0.9% NaCl infused at 100 µL per hour. Pressure was calibrated after warming the catheter (Model SPR-838 Millar instruments, Houston, TX, USA) in 0.9% sodium chloride (NaCl) at 37 °C for 30 min. The right internal carotid was then identified and ligated cranially. A 2F miniaturized combined conductance catheter-micromanometer was inserted into the carotid artery to obtain aortic blood pressure, then advanced into the left ventricle until stable pressure volume (PV) loops was obtained [31]. The abdomen was then exposed, and the inferior vena cava and portal vein were identified. Elastic bands were placed around these vessels to allow rapid reduction in cardiac preload. All loops were obtained with the ventilator turned off for 5–10 s and the animal apnoeic. Data were then acquired under steady state conditions and during preload reduction with parallel conductance values, which was obtained by the injection of approximately 200 µL of 10% NaCl into the right atrium [32,33]. Calibration from Relative Volume Units (RVU) conductance signal to absolute volumes (in µL) was undertaken using a previously validated method of comparison to known volumes in Perspex wells [34,35]. Using the pressure conductance data, a range of functional parameters were then calculated (Millar analysis software PVAN 3.4). These included: end diastolic pressure (EDP), end systolic pressure (ESP), stroke volume (SV), the slope of the end diastolic pressure volume relationship (EDPVR), maximum and minimum dP/dt mmHg/sec, the time constant of the isovolumic-pressure decline (τ method of Weiss) [36], the slope of the preload recruitable stroke work relationship (PRSW) [37], defined as the relationship between stroke work (SW) and end diastolic volume (EDV), where stroke work was the pressure-volume loop area for each beat.

### 2.9. Mitochondrial Respiration and ROS Studies

In a separate group of non-diabetic mice (N = 4/group) cardiac and renal mitochondria were isolated by differential centrifugation, as previously described [38]. Briefly, mice were euthanized by cervical dislocation, and the heart, and kidneys were dissected and placed on ice in Solution A (220 mM Mannitol, 70 mM Sucrose, 20 mM HEPES, 2 mM Tris and 1 mM EDTA. pH 7.2). The tissue was diced with scissors and washed three times in 10-times the volume of the wet weight of tissue with Solution A + 0.4% (*w*/*v*) BSA. The tissues were mechanically minced with a Tissue Tearor Homogenizer (John Morris) for 5 s. Tissue was further homogenized in a Weaton glass Dounce homogenizer with four passes of the loose pestle (120 µm clearance) then eight passes of a tight pestle (50 µm clearance) to break open the cells and release the cell contents. The homogenate was then centrifuged at 3000× *g* for 1.5 min to clear extracellular material, larger plasma membrane sheets and nuclei. The pellet was discarded and the supernatant, containing cell organelles retained. This centrifugation step was repeated until only minimal material pelleted. The supernatant was then centrifuged at 17,500× *g* for 2.5 min and the supernatant discarded. The pellet was then resuspended in 10 mL of Solution B (220 mM Mannitol, 70 mM Sucrose, 10 mM Tris and 1 mM EDTA. pH 7.2). The mitochondrial rich suspension was then centrifuged at 17,500× *g* for 4.5 min. The mitochondrial pellet was then resuspended in Solution B at a ratio of 1.5 mL per 1 g of original tissue weight. The crude mitochondrial preparation was then aliquoted and stored on ice to be used immediately in respiration experiments or stored at −80 °C for further biochemical analysis.

Mitochondrial ADP-stimulated respiration was measured using the Oxygraph 2 high-resolution oximeter (Oroboros Instruments, Innsbruck, Austria), using both Complex I and Complex II-linked substrates [38]. For each assay, 200–500 μg of mitochondrial protein was added to Miro3 buffer (200 mM sucrose, 20 mM taurine, 20 mM HEPES, 10 mM KH_2_PO_4_, 3 mM MgCl_2_, 3 mM EGTA, 1 g/L fatty acid free BSA; pH 7.1, 37 °C). To measure Complex I-driven respiration, substrates used were malate (final concentration of 2 mM) and pyruvate (5 mM). To measure respiration through Complex II, succinate (final concentration of 10 mM), was used as a substrate. With succinate, rotenone was added to block electron transfer though Complex I. To initiate State 3 respiration, 125 nmoles ADP was added to each chamber. The rate of oxygen consumption was monitored until the excess ADP was consumed and respiration returned to the basal rate (State 4).

Mitochondrial ROS production was measured in renal mitochondria using the DCFDA assay for hydroperoxide, as previously described [39].

### 2.10. Measurement of Kidney Malondialdehyde (MDA) Levels

MDA levels were measured as per manufacturer’s protocol (MAK085-1KT Sigma-Aldrich, St. Louis, MO, USA) with minor modifications. Mouse kidney homogenates were sonicated followed by lysis using MDA lysis buffer. Protein amount was estimated using BCA (Pierce^TM^ BCA Protein Assay Kit). A total of 200 µL of homogenized supernatant samples were transferred into fresh tubes. MDA-TBA adduct was formed by adding 600 µL of TBA solution to samples and standard vials. Samples were incubated at 95 °C for 60 min then brought to room temperature for 10 min. In total, 200 µL of samples and standard were transferred to 96-well plate and the plate read for absorbance at 532 nm using a TECAN SPARK 20M plate reader. The lipid peroxidation product was measured by the estimation of malondialdehyde (MDA). It was estimated by the reaction of MDA with thiobarbituric acid (TBA) to form a colorimetric (532 nm) product, which represents the amount of MDA present in the sample. The quantity of MDA in nmole in the samples were calculated using a standard curve.

### 2.11. Statistics

Adequate statistical power to detect biologically significant differences and to provide sufficient tissue for both structural and biochemical analyses was calculated as follows: with a minimal detectable difference of at least 30% and inter-animal variation of 25% (standard power of 80% and two-sided alpha of 0.05), sample sizes for each procedure described were eight. Data were managed in MS Excel (Version 2312 Build 16.0.17126.20132) and analyzed using Statistica for Windows (Version 13.3, Tibco, Palo Alto, CA, USA) software. Descriptive statistics, Pearson correlation coefficients and ANOVA with Fisher least significant difference (LSD) post hoc test used as appropriate, with significance taken at *p* < 0.05.

## 3. Results

**Figure 1:**
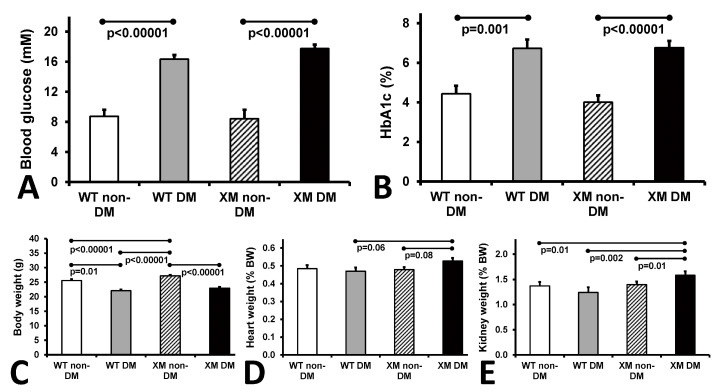
Animal characteristics. Measurements of glycemia. (**A**). Blood glucose levels of the four mouse groups on the day of sacrifice. The bars represent the concentrations of glucose expressed as mean values ± standard error of the mean of the group of 10 animals. (**B**). Levels of HbA1c of the four mouse groups on the day of sacrifice. The bars represent the percentage of glycated HbA1c as mean values ± standard error of the mean of the group of 10 animals. (**C**). Measurements of body weights. Body mass of the four mouse groups on the day of sacrifice. The bars represent the weights of the mice expressed as mean values ± standard error of the mean of the group of 10 animals. (**D**). Measurements of heart weight, and (**E**). kidney weight of the four mouse groups expressed as percentage of the body weight. The bars represent the weights as mean values ± standard error of the mean of the group of 10 animals.

Characteristics of the four animal groups are presented in Figure 1. Diabetic animals were lighter and had higher blood glucose and HbA1c levels than their non-diabetic counterparts. Diabetic xenomice had higher heart and kidney weights than WT mice, while WT mouse organ weights were not significantly altered by diabetes.

**Figure 2:**
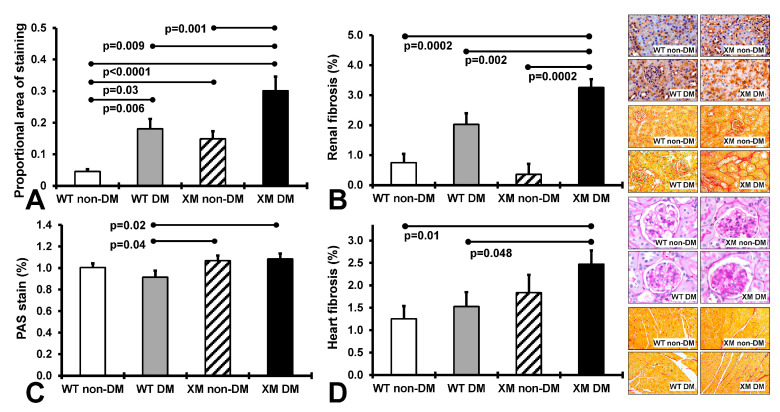
Measurements of renal and cardiac oxidative stress and fibrosis. (**A**). The levels of 3-nitrotyrosine in renal tissues obtained by quantification of the positive immunostaining. The bars represent the proportional areas stained positive for 3-nitrotyrosine expressed as mean values ± standard error of the mean of 10 animals. Representative images for the localization of 3-nitrotyrosine in renal tissues, as assessed by the proportional area of positive immunostaining in the 4 groups of mice are shown in the top image panel. (**B**). Measurements of renal interstitial fibrosis. The levels of intertubular interstitial collagen in renal tissues obtained by quantification of the positive pircrosirus red staining. The bars represent the proportional area stained positive for intertubular interstitial collagen expressed as mean values ± standard error of the mean of 10 animals. Representative images for the localization of intertubular interstitial collagen fibers in renal tissues, are shown in the second from top image panel. (**C**). Measurements of renal glomerular sclerosis, as assessed by the grading of periodic acid-Schiff (PAS) staining of the glomeruli in the 4 groups of mice. The bars represent the levels of glomerular sclerosis expressed as mean values ± standard error of the mean of 10 animals. Representative images for the level of glomerulosclerosis in renal tissues are shown in the image panel third from top. (**D**). Measurements of cardiac interstitial fibrosis. The levels of interstitial collagen in cardiac tissues obtained by quantification of the positive pircrosirus red staining. The bars represent the proportional area stained positive for intertubular interstitial collagen expressed as mean values ± standard error of the mean of 10 animals. Representative images for the localization of interstitial collagen fibers in cardiac tissues are shown in the bottom image panel.

Renal and cardiac structure and oxidative stress. Figure 2 presents results of measures of renal nitrotyrosine staining, fibrosis, and glomerular sclerosis and cardiac fibrosis. Renal nitrositive stress was increased by diabetes in both WT and XM, being higher at baseline in XM and again higher than WT in the diabetic milieu. Renal fibrosis was also increased in XM by diabetes, and higher than WT. Glomerular sclerosis was increased in XM compared to WT with diabetes. Cardiac fibrosis trended higher with diabetes in both WT and XM, but was not significantly increased. It was significantly increased in XM vs. WT with diabetes.

**Figure 3:**
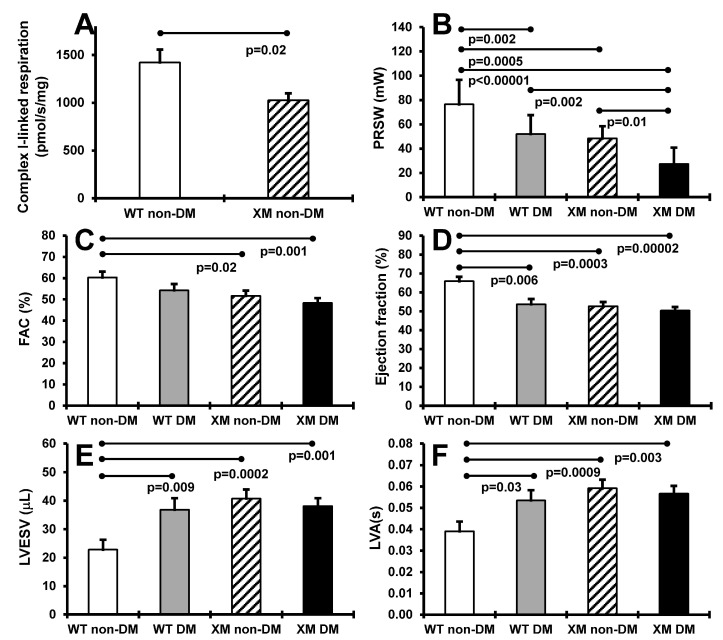
Measurements of cardiac systolic functions. (**A**). Maximal ADP-stimulated respiration rate in heart mitochondria from non-diabetic WT and XM. (**B**). Preload recruitable stroke work (PRSW) of the four mouse groups on the day of sacrifice. The bars represent the levels of PRSW in milliwalts (mW) expressed as mean values ± standard error of the mean of the group of 10 animals. (**C**). Left ventricular fractional area change (FAC) of the four mouse groups on the day of sacrifice. The bars represent the percentage of FAC as mean values ± standard error of the mean of the group of 10 animals. (**D**). The percentage of ejection fraction of the four mouse groups on the day of sacrifice. The bars represent the percentage of ejection fraction as mean values ± standard error of the mean of the group of 10 animals. (**E**). Left ventricular end-systolic volume (LVESV) of the four mouse groups on the day of sacrifice. The bars represent the levels of LVESV in microliter (μL) expressed as mean values ± standard error of the mean of the group of 10 animals. (**F**). Left ventricular activation time (LVA) of the four mouse groups on the day of sacrifice. The bars represent the LVA in seconds (s) as mean values ± standard error of the mean of the group of 10 animals.

Cardiac function. Figure 3 presents results of heart mitochondrial respiration in non-diabetic animals, and cardiac function assessment in both diabetic and non-diabetic animals. OXPHOS complex I-linked ADP-stimulated respiration was significantly decreased, by approximately 40%, in cardiac mitochondria from XM. Preload recruitable stroke work was significantly lower in XM compared to WT at baseline, and was decreased by diabetes in both mouse groups. Diabetic XM showed significantly lower values than diabetic WT mice. Left ventricular fractional area change trended lower, but was not lower in either mouse group with diabetes but was significantly lower in XM at baseline. Ejection fraction was significantly impaired by diabetes in WT mice; in XM, this measure was significantly lower than WT at baseline but not further lowered by diabetes. A similar result was obtained for left ventricular end-systolic volume, being higher at baseline in XM, and being increased only in WT diabetic mice.

**Figure 4:**
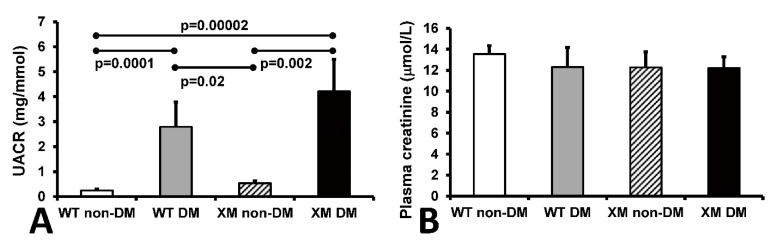
Measurements of renal function. Measurements of renal function. (**A**). Albumin to creatinine ratio (UACR) in mouse urine samples. The bars represent the UACR levels expressed as mean values ± standard error of the mean of 10 animals. (**B**). The concentrations of creatinine in mouse plasma samples. The bars represent the levels of plasma creatinine expressed as mean values ± standard error of the mean of 10 animals.

Renal function. Urine albumin/creatinine ratio (UACR) and plasma creatinine levels are shown in Figure 4. UACR was significantly increased in diabetic WT and XM groups, while plasma creatinine levels were not significantly different between any groups.

**Figure 5:**
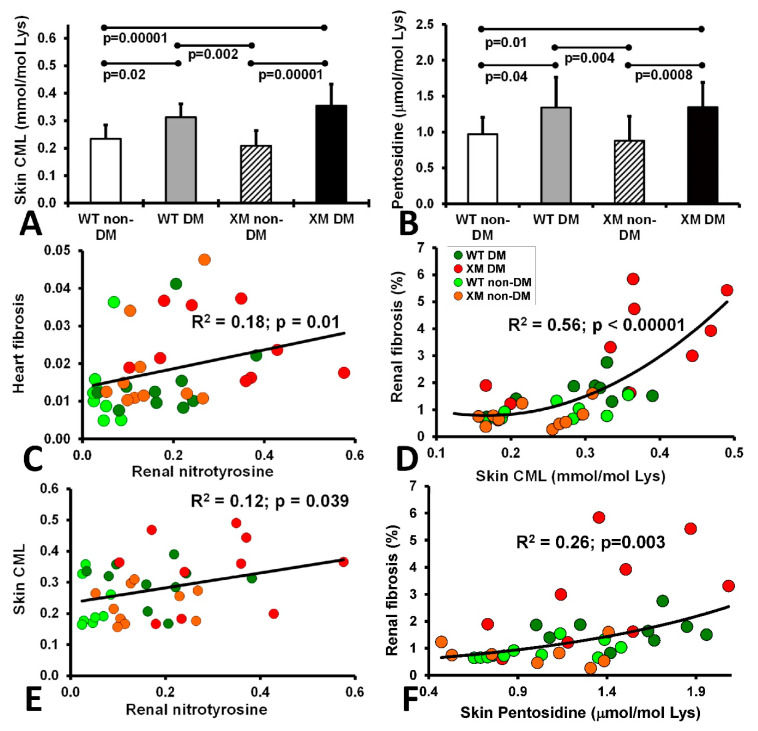
Measurements of skin AGEs and correlations of fibrosis, nitrotyrosine and AGEs. The level of CML (**A**) and Pentosidine (**B**) in the animal skin extracted from the 4 mouse groups upon sacrifice. CML level is expressed as mmol per mole of Lysine and Pentosidine level is expressed as ± mol per mole of Lysine. The bars represent the AGE levels as mean values ± standard error of the mean of the group of 10 animals (ANOVA - Bonferroni). (**C**). Correlation of heart fibrosis and renal nitrotyrosine. (**D**). Correlation of renal fibrosis and skin CML. (**E**). Corelation of skin CML and renal nitrotyrosine. (**F**). Correlation of renal fibrosis and skin pentosidine.

Oxidative stress markers. Skin carboxymethyllysine (CML) and pentosidine levels are shown in Figure 5, along with correlations between these AGE measures and various cardiac and renal oxidative stress and fibrosis measures. Both CML and pentosidine were increased in diabetic animals compared with non-diabetic animals, while the WT and XM groups did not show a significant difference. Significant correlations were evident between cardiac fibrosis and renal nitrotyrosine; between skin CML and renal nitrotyrosine, between skin CML and renal fibrosis, and between renal fibrosis and skin pentosidine.

**Figure 6:**
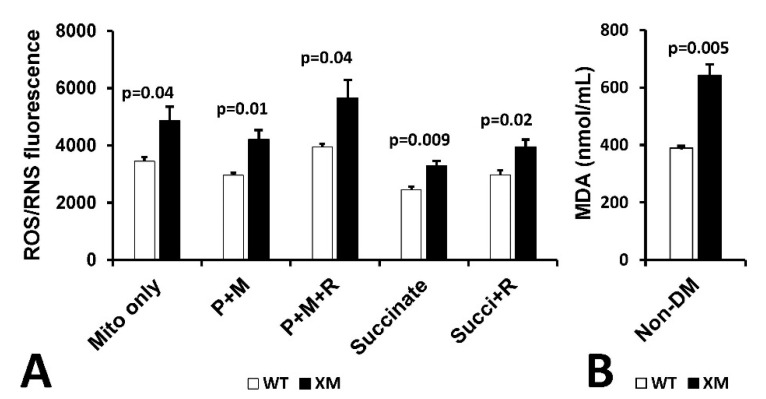
Evidence of Oxidative Stress in kidney of xenomitochondrial mice at baseline. DCFDA fluorescence detection of H_2_O_2_/RNS production (**A**). Isolated kidney mitochondria were incubated alone (mito only); with pyruvate + malate for complex I-linked oxidation (P + M), with pyruvate + malate + rotenone (P + M + R); with succinate for complex II-linked oxidation; and with succinate + rotenone (Succi + R). Kidney MDA levels (**B**).

Mitochondrial reactive oxygen/reactive nitrogen (ROS/RNS) production and malondialdehyde (MDA) in kidney. Figure 6A shows mitochondrial H_2_O_2_/RNS production as measured by DCFDA in mitochondria isolated from kidney of non-diabetic WT and XM mice. Increased ROS/RNS production was observed in XM compared to WT in all conditions used. Figure 6B shows results of measurement of MDA levels in kidney homogenates from non-diabetic WT and XM, showing elevated MDA levels in the XM kidney.

## 4. Discussion

In an oxidative stress model, including relevant diabetic and non-diabetic control groups, we tested the effects of insulin-treated diabetes in a novel mouse model of mitochondrial dysfunction. We demonstrated diabetes-related exacerbation of cardiac and renal dysfunction, increased organ fibrosis, tissue AGEs and oxidative stress, including renal mitochondrial oxidative stress which were greatest in the diabetic xenonitochondrial mouse (XM). There were significant correlations between renal nitro-tyrosine and skin AGEs and cardiac fibrosis.

Mitochondrial dysfunction is implicated in diabetes microvascular (retinal [13], kidney [12] and nerve) complications and in macrovascular complications, including cardiovascular disease and heart dysfunction [8,12], hence is an ongoing research focus in diabetic complication pathogenesis. The C57BL/6 mouse is the most widely used for many pre-clinical studies but is not regarded as the best for modelling diabetic complications [40]. Better models that develop more fulminant diabetic complications would be a valuable tool for research, including pre-clinical studies of potential interventions. Here, we present evidence that by altering the mtDNA of the C57BL/6 mouse, worsened diabetes complications, related to mild respiratory chain impairment and consequent increased oxidative stress.

Until the last few thousand years, human populations existed as relatively isolated groups following the earlier migrations out of Africa [41]. This allowed the co-evolution of nuclear and mtDNA OXPHOS genes for optimal function in vastly different environments [41]. MtDNA is exclusively maternally inherited and several ancient single nucleotide variants are inherited as a group, termed a haplogroup. MtDNA haplogroups represent human populations having the same ancestral origins and migration patterns and have been associated with disease risk [42,43].

The xenomitochondrial mouse harbors the mtDNA from an Indian native mouse (*Mus terricolor*) on a *Mus domesticus* (C57BL/6J) background, which results in 155 amino-acid substitutions in mtDNA-encoded OXPHOS genes compared with *Mus domesticus* [19,21]. Mitochondrial DNA diversity has been completely lacking in inbred laboratory mouse strains due to the historical accident of introgression of a single maternal mtDNA into all commonly used mouse strains [44,45]. For this reason, the potential contribution of mtDNA variation has not been explored in mouse models of diabetes. Where investigated, mtDNA haplogroup associations have been found in human populations with regard to diabetes complication risk [46,47,48,49]. Our XM mouse model incorporates mtDNA haplotype diversity into mouse models of diabetes. This is achieved by mirroring the diversity of mtDNA haplogroups observed in human populations with inter-specific mtDNA differences. The regions of mtDNA that vary between human haplogroups are reflected in the diversity among closely related mouse species, exemplified by the *Mus terricolor* mouse, which serves as an mtDNA donor in our model.

Transmitochondrial mice using natural variants are a valuable tool for studies investigating mtDNA effects in disease. A previous report used a transmitochondrial mouse harboring a single point mutation in the mtDNA ND6 gene, which resulted in increased lymphoma development [50]. This study did not investigate diabetes development in the model, showing only that older mice under glucose challenge were slower to clear the glucose, but baseline glucose and insulin levels were not altered. Other reports from Ballinger’s group used pronuclear transfer to introduce mtDNAs from different mouse strains [51,52], showing altered insulin sensitivity and glucose metabolism consequent to the mtDNA differences, but diabetes complications were not studied [53].

As expected, the XM had impaired mitochondrial function and increased reactive oxygen species generation in cardiac and renal tissues and increased nitrotyrosine staining and the lipid peroxidation marker of oxidative stress malondialdehyde in renal tissue at baseline, which was exacerbated by the presence of diabetes. AGEs are implicated in the pathogenesis of diabetic micro- and macrovascular complications [2]. Carbonyl stress plays a crucial role in the formation of AGEs. The term “carbonyl stress” refers to an imbalance between the production of reactive carbonyl species (RCS), such as methylglyoxal and glyoxal, and the cellular defense mechanisms that neutralize these toxic compounds. Excessive levels of RCS lead to protein, lipid, and nucleic acid modifications, fostering the generation of AGEs. The carbonylation of biomolecules by reactive carbonyls induces structural alterations and functional impairments, contributing to the pathophysiology of diseases. AGEs, in turn, exacerbate oxidative stress and inflammation [54,55].

A tissue which can be used as a good long-term measure of glycation and oxidative stress is tissue collagen; e.g., skin AGEs, which in humans are usually increased in diabetes per se, and also in kidney damage, and are associated with and predictive of long-term complications, including advanced kidney disease and mortality [56,57]. Abundant AGEs are CML and pentosidine, which we quantified using gold-standard biochemical techniques, were found to be significantly increased in both diabetic vs. non-diabetic mouse groups and significantly higher in the diabetic vs. non-diabetic XM. Skin CML also correlated positively with renal nitrotyosine levels.

As expected, diabetes in both the WT and XM mice groups was associated with hyperglycemia (blood glucose and HbA1c) levels and lower body weight, with no significant difference between the WT and XM diabetic groups. Heart weight did not differ significantly between groups, but cardiac fibrosis was significantly greater in the diabetic XM than both the non-diabetic and diabetic WT mice. Various elements of echocardiographically assessed cardiac dysfunction were evident in both the non-diabetic and diabetic XM groups, sometimes being worse in the diabetic than non-diabetic XM group. The heart has a high-energy requirement, and in cardiac myocytes, mitochondria occupy approximately one third of the cell volume [58]. Cardiac damage in the non-diabetic XM is in keeping with cardiac damage and cardiomyopathy in humans with mitochondrial disorders [59].

Kidney weight, which is usually increased in people and animals with diabetes, was increased in both diabetic groups, as expected, and renal fibrosis and renal mitochondrial oxidative stress markers were increased in the diabetes groups, more so in the XM group. The kidney is also a high energy demand organ, and mitochondrial damage is implicated in diabetic nephropathy, including tubulopathy, which is usually reflected by increased albuminuria, which was evident in the diabetic XM [10,60,61]. The diabetic XM also exhibits markedly increased renal and glomerular fibrosis, in keeping with likely renal filtration damage. Perhaps with longer diabetes duration or addition of other more detailed measures of renal function, plasma creatinine differences may have been observed between our study groups of non-diabetic and diabetic mice.

## 5. Study Strengths and Limitations

Study strengths include the use of a novel mouse model with mitochondrial dysfunction, which as in human mitochondrial disease has mild cardiac dysfunction even in the absence of diabetes [59,62]. In addition, we used this model to test if diabetes tissue damage is aggravated by mitochondrial dysfunction. The diabetic mice were insulin-treated, as are humans with Type 1 diabetes, avoiding the potential confounder of non-insulin treatment. Animals were characterized in detail, using well-characterized techniques including their renal and cardiac structure and function, and assessments of tissue AGEs. 

Study limitations include the potential that findings in animal models may not translate into the human condition. There was renal dysfunction in the diabetic animals as reflected by increased albuminuria, which was highest in the XM diabetic mice, but plasma creatinine was not decreased. Longer duration of diabetes and other measure of renal filtration function such as creatinine clearance or Glomerular Filtration Rates (GFR) or cystatin C are of interest in future studies. Whilst mitochondrial dysfunction is implicated in the pathogenesis of diabetic retinopathy, and other mitochondrial disorders have retinal damage [63], retinae were not evaluated herein due to small eye size in mice and lack of the required experience and tools to conduct these analyses. No therapeutic interventions were tested, but given the study results provided herein, we believe this model may be useful for the preclinical testing of potential therapeutics. 

## 6. Conclusions

In summary, we demonstrate several novel important measures of diabetic tissue damage in the heart, kidneys and skin in mouse models which are exacerbated by variation in the mitochondrial genome. Our results implicate mtDNA variation as a new genetic factor to investigate in human diabetes, adding to recent human studies showing associations between mt-DNA-CN with diabetes per se and chronic diabetes complications [16]. This presents a unique new model to further research into the pathogenesis, prevention and treatment of chronic diabetes complications that are a cause of major morbidity and premature mortality. We suggest that preclinical studies of repurposed mitochondria active drugs, such as metformin and coenzyme-Q, and specific mitochondria targeting drugs [63,64,65,66,67] using the diabetic XM model are merited, followed by, if positive, human studies. 

## Data Availability

Data are contained within the article.
